# Effect of graphene oxide on modifying polyethersulfone membrane performance and its application in wastewater treatment

**DOI:** 10.1038/s41598-020-58472-y

**Published:** 2020-02-06

**Authors:** Azam Marjani, Ali Taghvaie Nakhjiri, Maryam Adimi, Hassan Fathinejad Jirandehi, Saeed Shirazian

**Affiliations:** 1 Department of Chemistry, Arak Branch, Islamic Azad University, Arak, Iran; 2 Department of Chemical Engineering, Science and Research Branch, Islamic Azad University, Tehran, Iran; 3 Department of Chemical Engineering, Farahan Branch, Islamic Azad University, Farahan, Iran; 4 Department of Chemistry, Farahan Branch, Islamic Azad University, Farahan, Iran; 5 Department for Management of Science and Technology Development, Ton Duc Thang University, Ho Chi Minh City, Viet Nam; 6 Faculty of Applied Sciences, Ton Duc Thang University, Ho Chi Minh City, Viet Nam

**Keywords:** Membranes, Theoretical particle physics, Environmental chemistry, Chemical modification

## Abstract

In the present paper, Graphene Oxide (GO) particles were prepared via Hummer method, and used in synthesis of composite membranes. Polyethersulfone (PES) nanocomposite membranes were synthesized via wet phase inversion technique, and using water as non-solvent. The membrane morphology was investigated using scanning electron microscopy (SEM). Change in the membrane surface hydrophilicity after modification was studied using contact angle measurements. The performance of fabricated PES nanocomposite membranes was measured by evaluating pure water flux, salt rejection, dye retention and heavy metals removal. The results indicated that by increasing the filler percentage up to 5 wt.%, the contact angle between the water droplet and the membrane surface was decreased and the droplet was more dispersed on the membrane surface which implies higher hydrophilicity of the prepared nanocomposite membranes. Moreover, the experimental results corroborated that addition of GO to the membrane significantly improved the pure water flux, salt rejection and heavy metals removal, and can be used as a novel methodology for preparation of high performance membranes in water treatment.

## Introduction

Over the last decades, substantial development in chemical industries has eventuated in an increment in the processing speed and a reduction in energy consumption^1^. It has been recognized that separation and purification of different materials is one of the most significant techniques in chemical and biochemical engineering which has major contribution to total processing costs^2–6^. In order to implement the industrial processes, raw material components must be separated and the obtained products should be purified as well. On the other hand, in the majority of chemical industries, the requirement of separation procedures seems to be unavoidable to efficiently manage the deleterious impressions of greenhouse gases on environment^7–9^. In this regard, membranes have been developed for the efficient separation and purification of various types of materials in solid, liquid and gas states. Although the membrane separation procedure is more recent than distillation, adsorption, crystallization and liquid-liquid extraction, significant advancements have been observed in its application over the past two decades due to the efficiency and ease of operation^4,5,10–17^.

Both polymeric and inorganic membranes have been developed for the purpose of separation and reaction. Polysulfone-based membranes such as Polyethersulfone (PES) are utilized for the fabrication of nanofiltration membranes because of their outstanding mechanical/thermal resistance, chemical compatibility and stability over an extensive range of pH^18^. The main disadvantage of the PES membranes is its intrinsic hydrophobic nature. Due to the absorption of organic impurities, these membranes are susceptible to fouling and blockage which leads to decreasing their life span^19^. In order to optimize the performance of nanofiltration membranes, they should be modified prior to usage in separation. Increasing the membrane’s hydrophilicity is regarded as a good option to improve membrane performance. Indeed, the modification techniques lead to a compromise between the hydrophilicity and hydrophobicity nature of polymeric membranes, and improves the separation properties of membranes in removal of ions and organic molecules from aqueous solutions^20^.

In recent years, one of the substances applied to remove contaminants and impurities from the water is Graphene Oxide (GO) and its modified derivatives, which absorb suspended and water-soluble materials due to its high surface area and functional groups. The first and most important investigation on membrane fabrication incorporated with GO particles was reported by Nair *et al*.^21^. Further investigations were implemented by Cohen *et al*., in which the ability of nanoporous Graphene for salt rejection in a reverse osmosis membrane has been simulated^22^. Zhang *et al*. studied the GO effect on the phase inversion process of the coagulation bath on morphology, surface properties, mechanical strength, and separation properties of prepared membranes. The results proved that increment in the amount of GO in the membrane structure significantly enhanced the amount of flux passing through the membrane, the mechanical strength and the separation properties^1^. Zeinadini *et al*. evaluated the performance of nanofiltration membranes using GO nanoparticles. In their study, the effect of GO nanoparticles on the morphology and anti-fouling properties of membranes was investigated. The results illustrated that by adding the GO to the membrane matrix structure, the amount of flux was increased. Moreover, the excellent dye removal from industrial effluents has been obtained applying this type of membrane^23^.

Ganesh *et al*. prepared polysulfone mixed matrix membranes using graphene dispersion via fuzzy separation technique. The difference in hydrophilicity was studied by measuring the surface wettability and water swelling. The contact angle data demonstrated that the addition of GO to the membrane surface would increase the surface hydrophilicity of prepared membranes^24^. Ionita *et al*. prepared polysulfone-GO nanocomposite and studied its structure, surface properties and mechanical/thermal performances. The analyses showed great compatibility and excellent dispersion of the polysulfone polymer matrix that was observed even for a very low amount of GO^25^. Zhang *et al*. studied preparation of GO composite membranes for separation of pollutants from water. They fabricated membranes with specific adsorption characteristics by blending approach. The prepared membranes were used as adsorbent for removal of cationic and anionic dyes. They reported that the GO composite polymeric membranes are great candidate for wastewater filtration^26^.

The main objectives of this paper are to experimentally synthesize the GO with modified Hummers method, prepare the polymer membranes incorporated with GO particles using phase inversion method and investigate the prepared Polyethersulfone nanocomposite membrane performance including pure water flux, salt rejection, dye rejection, removal of heavy metals and fouling. Additionally, the membrane morphology/hydrophilicity are studied using scanning electron microscopy (SEM) and contact angle experiments, respectively.

## Experimental and Methodology

### Synthesis of graphene oxide (GO)

Hummer method was used for synthesis of GO particles^27^. 5 gr of graphite powder (Fluka) was slowly added to a mixture of 100 ml sulfuric acid (Merck) and 12 ml phosphoric acid (Merck), which was placed in ice bath under a magnetic stirrer. Then 2.5 gr of ammonium nitrate was added to this mixture during 30 min. After that, 20 gr of potassium permanganate powder (Merck) was gradually added to the mixture over 60 min, so that the temperature of the mixture should not be higher than 5 °C. After addition of the abovementioned materials, the mixture was stirred at a temperature below 5 °C for another 120 min. At this stage, the color of the mixture turns to black. Then, using an electric heater, the temperature of the mixture was increased to 40 °C and consequently the mixture was homogeneous using an electrical stirrer for 1 hr. At this point the color of the slurry becomes green. The temperature of this set was then increased to 98 °C and stirred for 60 min. At the end of this period, the obtained solution was added dropwise to 400 ml frozen distilled water and after 5 min, 15 ml of oxygenated water (Merck) was added. Finally, the solution was centrifuged and washed 4 times with deionized water and then washed once with hydrochloric acid 5% (Merck) and again, this procedure was repeated twice with deionized water. The sample was vacuum-dried in an oven at 60 °C for 24 hrs.

### Preparation of the PES/GO nanocomposite membranes

Polyethersulfone (PES, BASF Co.) was used for preparation of membranes via phase inversion method. Different percentages of the filler (1, 3, 5 wt.%) relative to the polymer matrix weight were selected and the nanocomposite membranes were fabricated for each of the filler percentage. As an example, a nanocomposite membrane with 1 wt.% of filler is prepared using the following procedure:

0.2 gr (1 wt.% relative to the PES polymer weight) of GO (as filler) was dispersed in 0.5 gr of N-Methyl-2-pyrrolidone (NMP, Merck) solvent and was added to a homogeneous mixture of 2 gr of polymer (PES) and 1 gr of polyethylene glycol (PEG) homogenized in 6.48 gr of NMP solvent (total weight of the mixture is 10 gr). The solution is then placed on a magnetic stirrer at 60 °C for 24 hr. After this period, the solution is cast using a suitable blade onto a perfectly flat glass and immersed in the coagulation bath (room temperature, anti-solvent water) for 30 s until the solvent displacement process and nanocomposite film formation occur (phase inversion process). For other fillers, the same procedure as described was used and the nanocomposite membrane is provided considering this difference that the weight of the selected solvent for the polymer mixture (PES + PEG) will vary for different percentages. Table 1 describes the applied compositions for preparing the nanocomposite membranes. In all cases, PEG is considered as the controlling agent of membrane pore size.

**Table 1:** Compositions used for preparing the nanocomposite membranes.

Sample	NMP solvent (%)	PES (%)	PEG (%)	Weight percent of filler (%)
M0	70	20	10	0
M1	69	20	10	1
M2	67	20	10	3
M3	65	20	10	5

### Permeation tests

**Figure 1:**
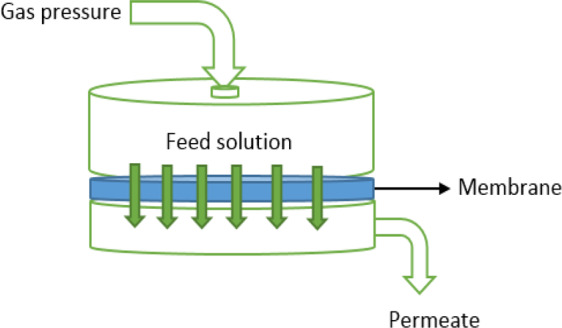
The nanofiltration cell used in the experiments.

In order to determine the hydrophilicity of the nanomembranes surface, the permeability test was applied. All permeability experiments were performed in a cell with dead-end mode to using circularly cut membranes with an effective surface area of 3.73 cm^2^. The applied external pressure is supplied by a nitrogen gas cylinder which is capable of generating pressure up to 12 bar. The membrane is first cut and inserted according to the size of the nanofiltration cell loading portion. As a feed solution, distilled water is used and the permeability test is performed by adjusting the external pressure between 1 to 7 bar for 1 min for each applied pressure. Then, from the volume of effluent, the effective membrane area, the operation time, net water flow is reported. The operation is performed for all prepared nanomembranes. Figure 1 illustrates the nanofiltration cell used in this work. For conducting the salt rejection experiments using the membrane samples, 0.01 M of NaCl, MgSO_4_ and Na_2_SO_4_ salt solutions were used. The applied operational pressure was 5 bar. The conductivity method was used to determine the concentration of samples passed through the loaded membranes inside the nanofiltration cell. All measurements have been conducted twice and the average values are reported throughout the paper. The variability was calculated to be less than 10% for all measurements.

The permeation flux is calculated as follows:

1 $$Flux=\frac{V}{A\,t}$$

where *V* is the volume of permeated solution (L), *A* is the membrane surface area (m^2^), and *t* is the experiment time (h).

### Dye and heavy metals removal tests

10 ppm of methylene blue (MB) and methyl orange (MO) solutions were applied to conduct the dye removal experiments using the prepared membrane samples. The applied operational pressure was 5 bar. The test time for each membrane sample was 60 seconds. UV spectroscopic method was used to determine the concentration of samples permeated through the membranes. In order to carry out the heavy metals removal experiments using membrane samples, 10 ppm ZnSO_4_.7H_2_O, 10 ppm CuSO_4_.5H_2_O and 2500 ppm Cd (NO_3_)_2_.4H_2_O salt solutions were used. The applied operational pressure was 5 bar. The test time for each membrane sample was 60 seconds. The conductivity method was utilized to determine the concentration of membrane-loaded samples in nanofiltration cells.

### Fouling tests

Bovine serum albumin (BSA) solution in 100 ppm phosphate buffer (PBS: 50 mM, pH:7.4) was used to perform the membrane fouling test. The test was performed in two modes, i.e. dynamic and static. In the dynamic mode, the nanofiltration cell was used for the test in which the membrane sample was cut to the appropriate size for the test. First, at a pressure of 5 bar for 60 s, the pure water flux was measured (Jw1). Then, the nanofiltration cell was filled with BSA solution in 100 ppm PBS, and the protein flux was measured at the same pressure and duration. Then, after washing the membrane, the pure water flux rate was again measured (Jw2) and flux recovery ratio (FRR) was reported for the membranes using the following equation:

2 $$FRR=\frac{Jw2}{Jw1}$$

This procedure was performed for all membrane samples and the results were reported. In the static type, the membrane samples were first cut in equal sizes with an effective surface area of 0.5 cm^2^ and the samples were immersed in phosphate buffer solution (PBS) for 2 hr. After this period, the samples were extracted from phosphate buffer solution and each sample was individually placed in test tubes filled with 5 ml of protein solution in a 30 °C water bath for 24 hr to reach equilibrium. The amount of adsorbed protein on the surface of each membrane was determined by comparing the absorption intensity of the protein solutions before and after the experiment. UV-vis spectroscopy (model 8453, Agilent, U.S.A) at 280 nm was used to measure the absorption intensity.

## Results and Discussion

### Investigation of synthesized graphite and graphene oxide IR spectra

**Figure 2:**
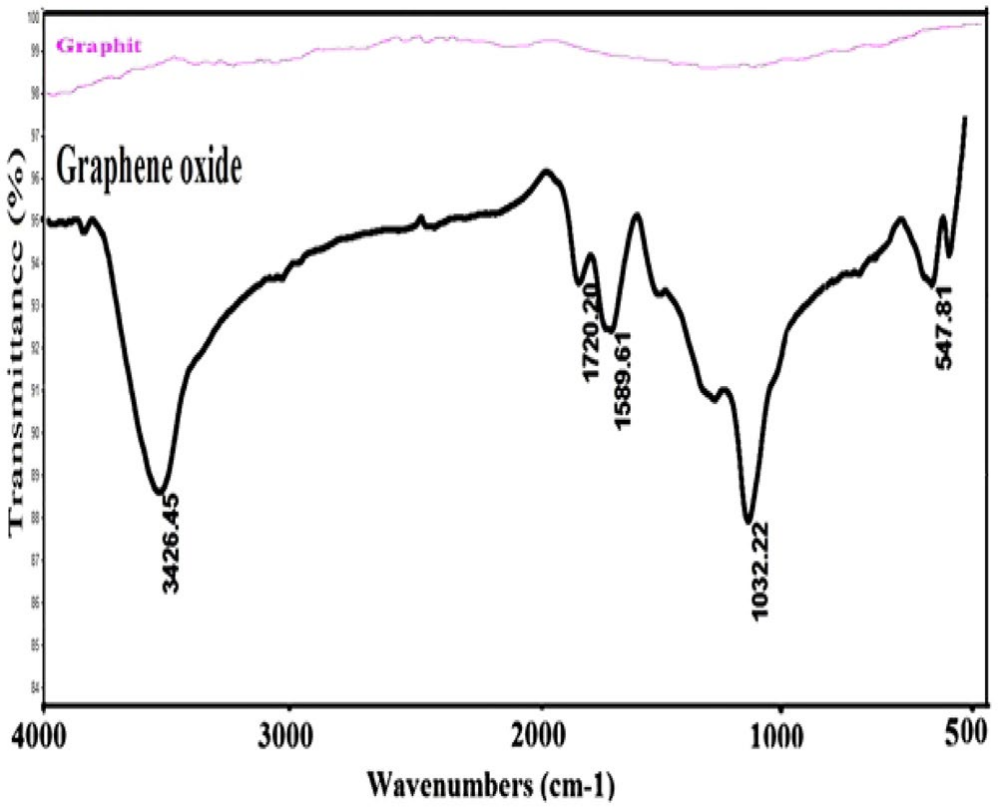
IR spectra of graphite and graphene oxide.

As can be seen in Fig. 2, the IR spectrum of GO indicates the presence of many factors on the graphite sheet after oxidation. A broad peak is seen above 3000 cm^−1^, which is justified due to the O-H stretching of the surface functional groups as well as absorption of water molecules on the GO layers. OH may exist in form of alcoholic, phenolic, and carboxylic acid. The strong peak at 3426 cm^−1^ corresponds to the O-H stretching of the GO surface groups and is confirmed by the strong band at 1032 cm^−1^ (related to the C-O stretch). The 1720 cm^−1^ band is associated with the C=O carboxyl group (COOH). The peak around 1589 cm^−1^ is related to the epoxide bond and the bending vibration of water molecules in the GO matrix, whereas the IR spectra of graphite indicate no specific properties, confirming that the graphite is successfully oxidized to GO by the applied synthesis method.

### TEM and SEM images of graphene oxide

**Figure 3:**
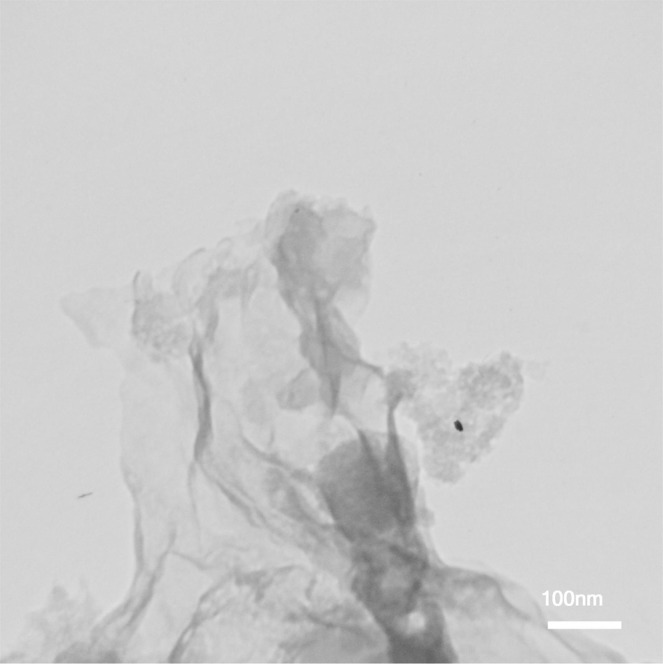
The TEM image of synthesized graphene oxide.

**Figure 4:**
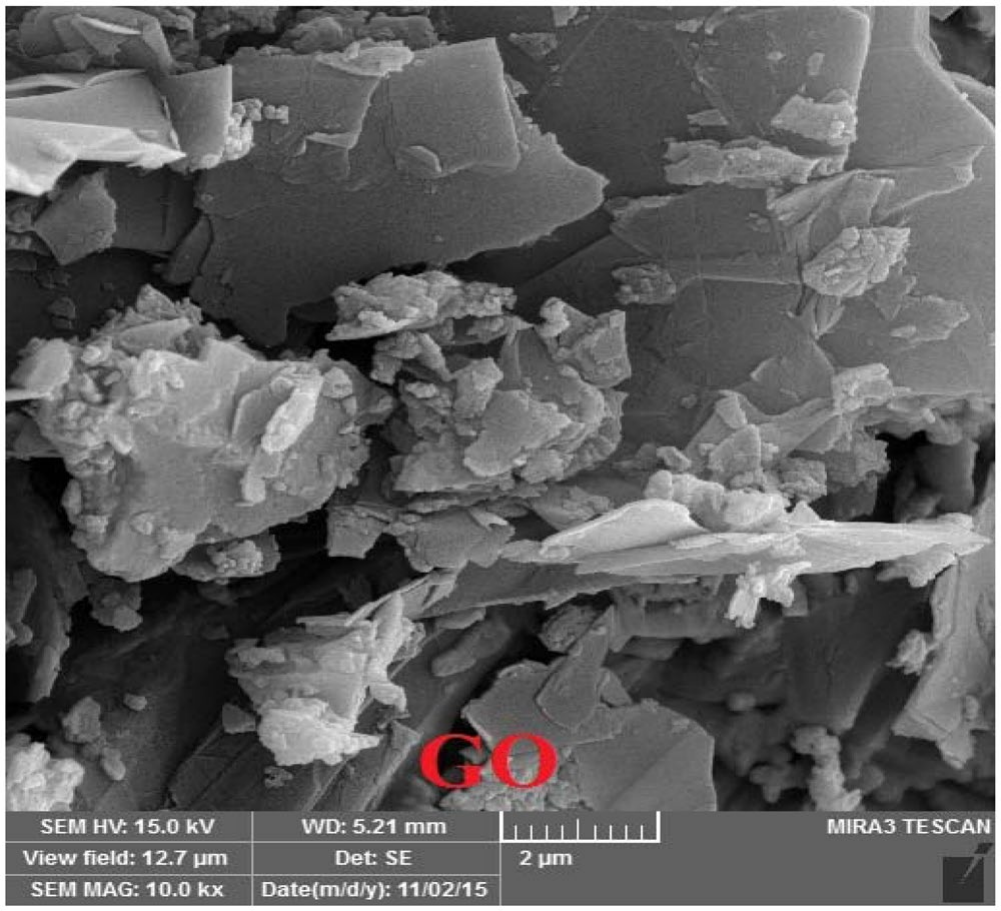
The SEM image of synthesized graphene oxide.

Transmission electron microscopy (TEM) is considered as it is an effective method for describing the morphology of GO. As illustrated in Fig. 3, the graphene oxide plates are seen as a flat surface. The TEM image confirms the correct and successful synthesis of GO nanostructure. Figure 4 illustrates the SEM images of graphene oxide. According to the image, it is observed that the plates are spaced apart and segregated in some places^19^.

### Investigation of graphite and graphene oxide XRD

**Figure 5:**
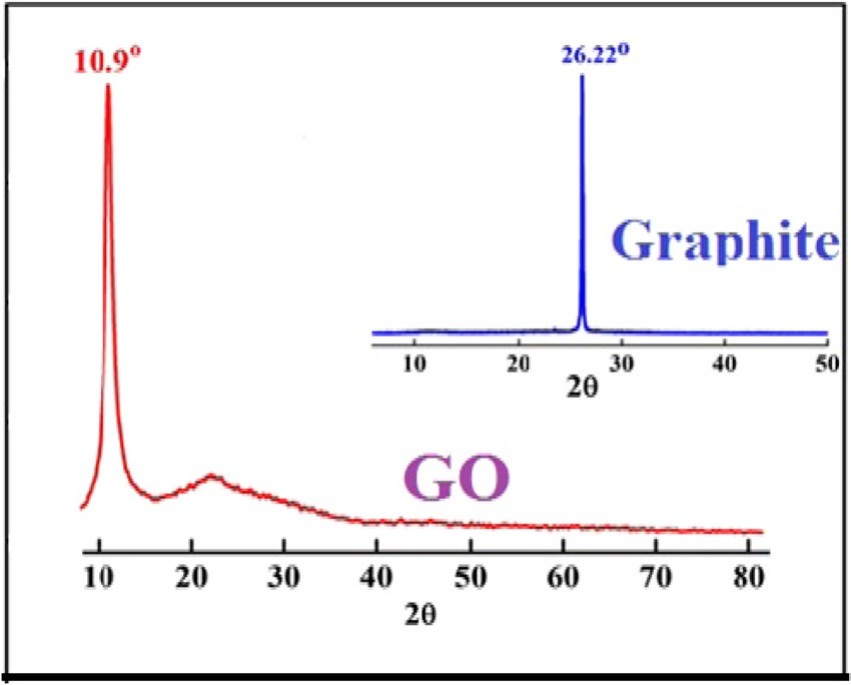
XRD spectra of graphite and graphene oxide.

Figure 5 obviously depicts the XRD pattern of graphite and graphene oxide. Based on the figure, for the graphene oxide, an intense peak is seen around 10.9° which is the characteristic peak of pure GO. In addition, comparison of the GO and graphite X-ray diffraction patterns corroborates that an area of the spectrum is out of alignment. Sharp branches appear at relatively high intensities which indicate the presence of more oxygenated functional groups caused by graphene oxide GO (increasing the number of oxygenated sites).

### Nanocomposite membranes surface morphology

**Figure 6:**
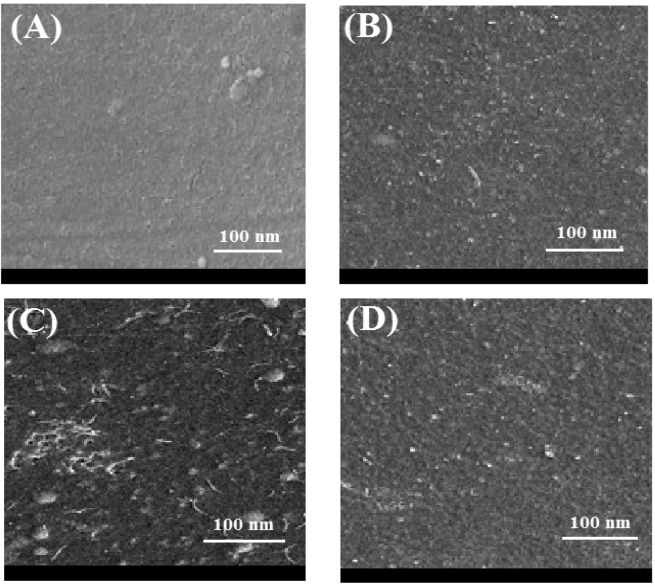
FESEM images from the surface of nanocomposite membranes. A:M0, B:M1, C:M2, D:M3.

**Figure 7:**
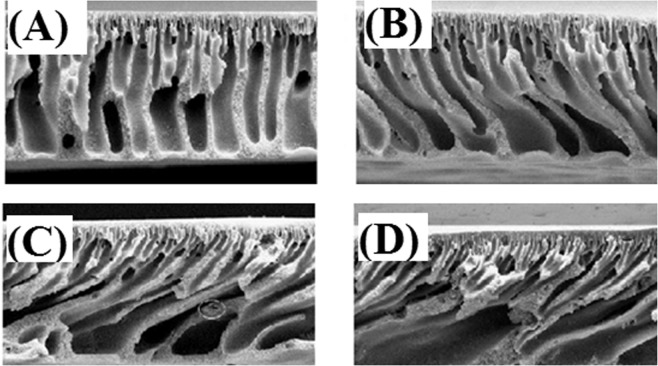
Cross-section FESEM images of prepared membranes.

The FESEM images of the fabricated membranes surface are shown in Fig. 6. It is clearly observed that the surface of PES unmodified membranes (A) are smoother than those membranes that are modified by different weight percentages of GO (B, C, D). Also, FESEM images of cross-section membranes are shown in Fig. 7. It can be seen that the presence of GO in the polymer matrix of the PES membranes has eventuated in the formation of more finger-like porous structure. The latter can be attributed to the enhancement of solvent replacement with nonsolvent (water) during phase inversion process, with increasing GO content which is of hydrophilic nature. It is also seen that the membranes surface was greatly smooth by the addition of GO particles, confirming well dispersion of GO in the polymer matrix. The FESEM images obtained from the membraness demonstrate that by increasing the GO content from 1 wt.% to 5 wt.%, the membrane porosity is increased.

### Nanocomposite membrane performance for modifying the pure water flux

**Figure 8:**
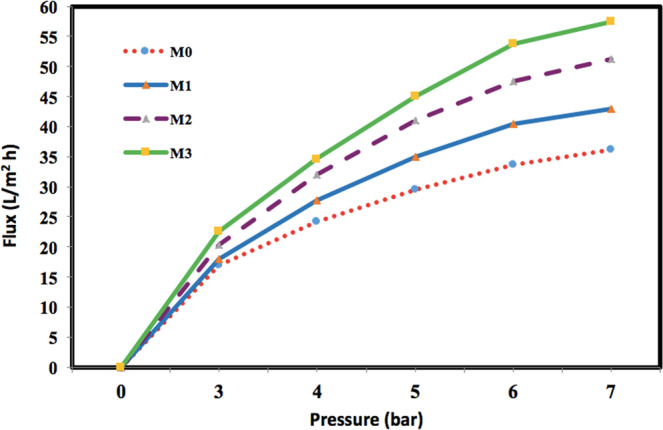
Water flux for GO-PES nanocomposite membranes with different filler percentages as function of pressure.

Figure 8 represents the obtained experimental data for the fabricated membranes with 0, 1, 3 and 5 wt.% GO filler content at different applied operational pressures. As seen, increase in the operational pressure caused a significant improvement in the water flux which is due to porous structure of the prepared membranes. Moreover, by increasing the filler content, the pure water flux is increased which is justified due to increasing the membrane hydrophilicity and porosity caused by increasing the amount of GO filler. As shown, increment in the applied operational pressure, the water flux for all membrane samples is increased. The figure illustrates that the highest amount of pure water flux is obtained for the nanocomposite membrane with 5 wt.% filler.

### GO-PES nanocomposite membrane performance in salt removal

Using the conductivity apparatus, the conductivity data of each sample of salt solution passed through the loaded membranes in nanofiltration cells were obtained during the rejection process. To obtain the concentration of salt samples passing through these membranes, standard solutions were prepared at lower concentrations than the feed solution and by drawing the calibration diagrams, the concentration of these samples were determined. The rejection percentage of different salts can be calculated using the following equation:

3 $$R\left( \% \right)=\left(1-\frac{{C}_{P}}{{C}_{a}}\right)\times 100$$

**Figure 9:**
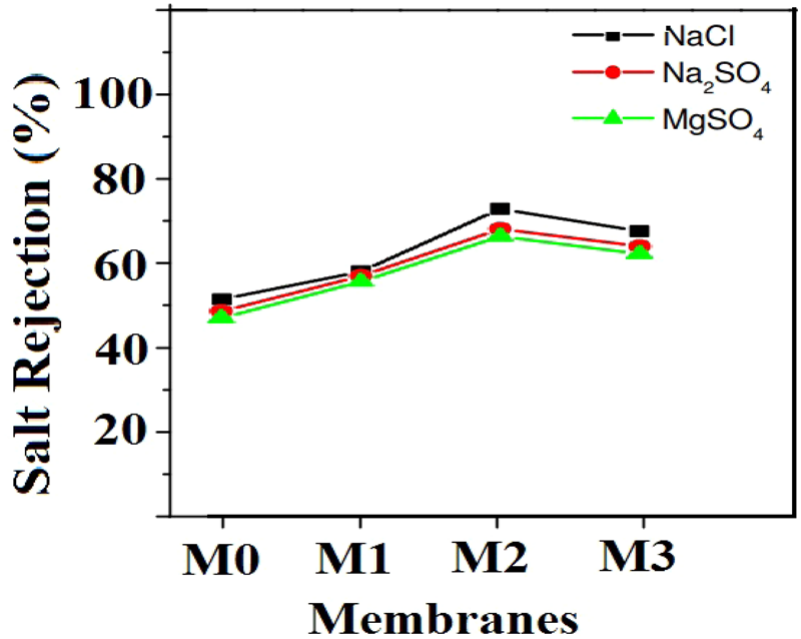
Salt rejection percentage for GO-PES nanocomposite membrane with different filler percentages.

Figure 9 demonstrates the salt rejection percentage for GO-PES nanocomposite membranes with filling percentages of 0, 1, 3 and 5 wt.%. As can be seen, increment of the filler percentage from 0 to 3 wt.% causes an increase in the amount of salt rejection, however the rejection decreases for the samples prepared with 5 wt.% of filler. This could be attributed to the appropriate distribution of GO particles which results in a better interaction with the ionic salt species. In other words, at optimum amount of filler (3 wt.%) GO particles are well dispersed in the polymeric matrix, and the number of active sites involved in the membrane surface increases, which eventuates in the highest rate of rejection. In the values above 3 wt.%, a slight decrease in the rate of rejection is observed which can be due to the accumulation of fillers on the membrane structure. Indeed, 3 wt.% filler can be considered as the optimum GO particles loading for preparation of nano-composite membranes. Moreover, the salt rejection results demonstrate that the highest percentage of rejection is obtained for NaCl salt and the lowest one is for MgSO_4_ salt, which could be attributed to the hydration size of ions for mass transfer through the membrane pores, ions diffusivity, and electrostatic interaction with the membrane.

### Evaluation of the GO-PES nanocomposite membrane performance for dye removal

**Figure 10:**
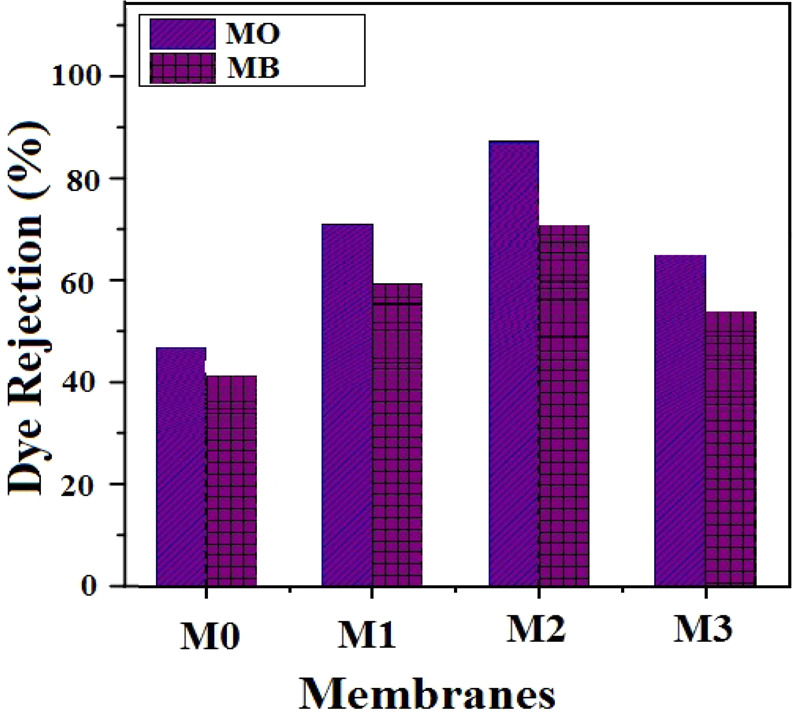
Dye rejection percentage of various GO-PES nanocomposite membranes with different filler percentages.

Using the spectrophotometer apparatus, the absorption data related to each of the dye solution samples passed through the membranes in the nanofiltration cells were obtained to measure the membrane rejection efficiency for dye. To achieve the concentration of dye samples passing through these membranes, standard solutions were prepared at concentrations lower than those of the feed solution and the concentration of these samples was determined by plotting calibration diagrams. The rejection percentages of MB and MO dyes for GO-PES nanocomposite membranes with filler percentages of 0, 1, 3 and 5 wt.% are illustrated in Fig. 10. To prevent the blockage of the nanocomposite membrane pores and increase the dye removal efficiency, the active surface and the initial layer of the nanocomposite membrane should be made of polymeric materials. These polymeric materials should have high ion charge density on the surface. Moreover, the ion charge type must be similar with the ion charge of the dyes present in the effluent so that the electrostatic repulsion between the dye molecules and the nanocomposite membrane reaches its maximum. It can be seen that the rejection of methyl orange (MO) dye in all range of filler percentages is higher than that of methylene blue (MB) dye. Since the prepared nanocomposite membrane surface has negative charge and considering the cationic feature of MO and MB and also the effect of Donan phenomenon (electrostatic repulsion), it can be said that the rejection of MO is more than MB^28^. The interaction between the dyes and the membrane surface can be electrostatic, π-π and covalent. Moreover, it is also seen that the best separation performance has been obtained for the samples with 3 wt.% of filler which is due to well distribution of particles.

### Heavy metals removal

**Figure 11:**
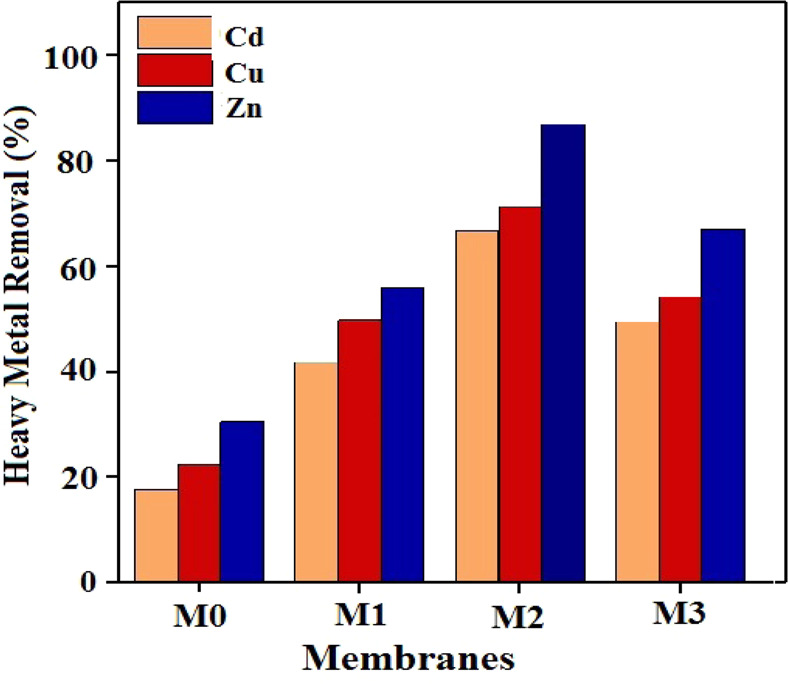
Percentage removal of zinc, cadmium and copper ions using various GO-PES nanocomposite membranes with different filler percentages.

The metal ions removal from aqueous solutions is attributed to two types of electrostatic and Van der Waals interactions. The electrostatic interaction is related to the surface charges created on the absorbent surface and Van der Waals interaction is associated to the functional groups coordination with the metal ions. In GO, the graphite carbon network with sp2 hybridization is strongly disrupted, and the carboxyl groups are placed on the edges. The presence of OH and COOH groups in GO enables the metal ions to be coordinated. Metal ions compete with each other for creating these interactions and behave differently in dealing with nanocomposites. Ultimately, metal ions are removed with a three-step mechanism involving the external diffussion of metall ions, diffussion into the nanocomposite and absorption in the nanocomposite. The removal percentage of different metals applying GO-PES nanocomposite membrane with filler percentages of 1, 3, and 5 wt.% is demonstrated in Fig. 11. As can be seen, by increasing the filler content to 3 wt.%, the removal percentages of zinc, cadmium and copper ions increase substantially and then decrease. Also, at the 3 wt.% of filler, the highest and the lowest removal percentage is related to zinc and cadmium metal ions, respectively.

### Analysis of the contact angle data for GO-PES nanocomposite membranes

**Figure 12:**
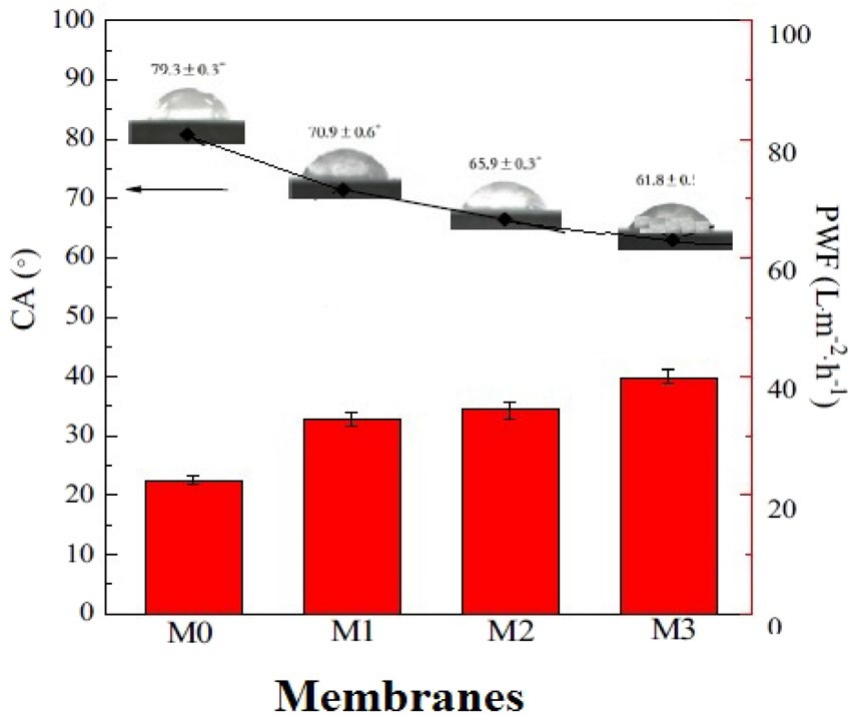
The schematic illustration of the contact angles between water droplets and GO-PES nanocomposite membrane surface for different filler percentages.

Figure 12 obviously demonstrates the contact angles between the water droplet and the surface of GO-PES nanocomposite membranes with the filler percentages of 0, 1, 3 and 5 wt.%. It is illustrated from the figure that as the filler percentage increases, the droplet is more dispersed on the membrane surface. In other words, by increasing the filler percentage, the hydrophilic nature of the membrane surface increases substantially.

### Analysis of membrane fouling

#### Dynamic tests

**Figure 13:**
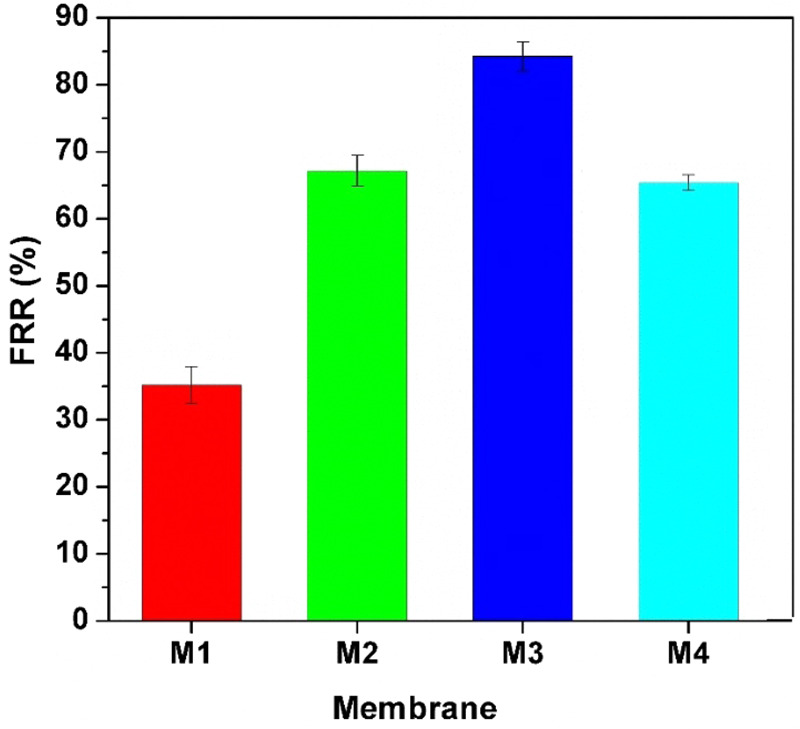
The results of dynamic fouling tests of GO-PES nanocomposite membranes. M1: neat membrane, M2: 1 wt.%, M3: 3 wt.%, M4: 5 wt.%. t = 60 s, pressure = 5 bar.

The dynamic test results of fouling for the prepared membranes are shown in Fig. 13. It is clearly seen that by increasing the percentage of GO in the membrane structure, FRR percentage is increased until 3 wt.% of GO, and then decreased for 5 wt.% filler. The reason could be due to aggregation of GO particles in the membrane pores at high loading which results in more fouling compared to other membrane samples.

#### Static tests

The results of static membrane fouling are reported in Table 2 in terms of adsorbed protein on the surface of membranes prepared with different filler percentages. It is seen that the highest amount of protein adsorption is for the neat polymeric membrane (with no filler), and the lowest is for the sample with 5 wt.% GO particles. The protein adsorption decreases with increasing the amount of filler which is attributed to the enhancement of membrane hydrophilicity with increasing filler percentage.

**Table 2:** Amount of adsorbed protein on the surface of membranes.

Membrane	Effective surface area (cm^2^)	Adsorbed protein on membrane (ppm/cm^2^)
Neat	0.5	87.48
1%	0.5	72.30
3%	0.5	55.20
5%	0.5	38.08

## Conclusions

In the present study, GO particles were applied to modify the PES membranes. GO was synthesized using the Hummer method, and charachterized by IR, TEM, and XRD analyses. The results proved that by modifying the membranes, their properties such as water flux, water absorption, hydrophilicity, and anti-fouling was improved considerably. By increasing the weight percent of GO filler from 0 to 3 wt.%, the rejection amount of different salts increases which is justified due to improving the membrane surface charge and its proper distribution. Increase in the amount of GO loading up to 3 wt.% improves the removal efficiency of heavy metals but increasing the GO to 5 wt.% slightly decreases the removal performance compared to the 3 wt.% sample. The reason for this phenomenon may be due to the accumulation of additives on the membrane surface, which makes pinhole on the membrane surface. It is well percieved that among the fabricated membranes, the membrane with 3 wt.% of GO as an additive performed better than the other membranes in separation.
