# Editorial Expression of Concern: Effect of graphene oxide on modifying polyethersulfone membrane performance and its application in wastewater treatment

**DOI:** 10.1038/s41598-024-59035-1

**Published:** 2024-04-23

**Authors:** Azam Marjani, Ali Taghvaie Nakhjiri, Maryam Adimi, Hassan Fathinejad Jirandehi, Saeed Shirazian

**Affiliations:** 1 Department of Chemistry, Arak Branch, Islamic Azad University, Arak, Iran; 2 Department of Chemical Engineering, Science and Research Branch, Islamic Azad University, Tehran, Iran; 3 Department of Chemical Engineering, Farahan Branch, Islamic Azad University, Farahan, Iran; 4 Department of Chemistry, Farahan Branch, Islamic Azad University, Farahan, Iran; 5 Department for Management of Science and Technology Development, Ton Duc Thang University, Ho Chi Minh City, Viet Nam; 6 Faculty of Applied Sciences, Ton Duc Thang University, Ho Chi Minh City, Viet Nam

Editorial Expression of Concern to: *Scientific Reports* 10.1038/s41598-020-58472-y, published online 06 February 2020

The Editors are issuing an Editorial Expression of Concern for this Article.

After publication, concerns were raised that Figure 7 of this Article appears to overlap with Figure 9 in a previously-published paper by different authors^1^. The Authors clarified that this data was used for comparative purposes only, and the citation omitted in error.

The Editors did, however, also request the original raw data underlying the Article, which the Authors are no longer able to provide. The Editors, therefore, want to alert the readers wishing to reproduce any of the results presented in this work that the underlying data is not available.

Saeed Shirazian did not explicitly state if they agree or disagree with this Editorial Expression of Concern. Azam Marjani, Ali Taghvaie Nakhjiri, and Maryam Adimi did not respond to the correspondence from Editors about this notice. The Editors were not able to obtain the current contact details for Hassan Fathinejad Jirandehi.
